# The Dental Convention and Men We Meet There

**Published:** 1923-07

**Authors:** Frank W. Sage


### THE DENTAL CONVENTION AND MEN WE MEET THERE

BY FRANK W. SAGE, D. D. S.

What great organizations our State Societies have become! Long years ago scores only attended where today we count hundreds. Less than forty years ago a lady dentist at a State Meeting attracted all eyes.

Hardly longer ago there was almost no system about conventions. Any dentist who could write a paper, any dentist who could not write a paper, was welcome to get up on the platform and read it, unannounced. The discussions were free-for-all affairs. No graces of oratory were required. If a dentist discovered after speaking a moment or two that he had said all he could think of respecting the subject proper, he might easily branch out in a tirade against the Goodrich Rubber Company for daring to presume to charge dentists for using their patents. It was by many regarded as a sneaking, underhand business to send agents to villages and towns all over the country, to spy out dentists secretly using the Company's patented methods without having paid the annual license. The subject was of perennial interest.

Still earlier it was the fashion to abuse amalgam, "nasty, fifthy amalgam." Cohesive gold for filling was just then coming in, and the idea was if there must be an alternative let it be tin foil, for the impecunious patient. A paper on tin foil was usually vociferously applauded. Yet tin foil would not stay in, excepting in the few favorable cases. It would not stand mastication. The occasion for applauding it seems to have been merely because it was not amalgam. Many went so far as to proclaim that any tooth worth filling at all was worth filling with gold. There is reason to suspect that even the most renowned operators in gold found a certain difficulty in "living up to their blue china" when it came to actual practice.

Today we hear nothing of "nasty amalgam." The prejudice against it has gone the way of the prejudice against rubber for plates. Funny bunch we dentists have always been.

Many there were who never attended dental societies or conventions. Most of these were of the somewhat elderly class of practitioners who liked to shut up shop about three P. M. and play checkers with the livery stable boss across the street until supper or perhaps bedtime. This dentist uses much amalgam, little gold, for fillings. Argues that amalgam in a front tooth is all right. Doesn't hold a polish like gold, to be sure. What of that? He argues that you can't always expect brilliancy in a filling any more than in a wife or sister. Isn't it saving the tooth? he challenges. In this burg, he goes on to say, patients are not too fussy about trifles. "I'm letting well enough alone," he concludes and calls back to the livery boss, "Fetch out the board Andy."

Some day this dentist is led by curiosity to go to a convention—a nearby one. Someone reads a paper on tin foil. Elderly dentist sniffs, whispers to another elderly dentist, a stranger, sitting beside him.

"Oh, no, does n't shrink, tin does n't. And does n't fill half the requirements. Fine fellow tin is, because he does n't shrink. Like praising a pickpocket because he does n't drink or chew. Huh!"

After all the philosophy of the old days was not so bad. It took account of the ease of the dentist, equally of the ease of the patient. Did not the dentist of that day know it was often a severe infliction on his patient to clasp a wisdom tooth and sometimes even dislocate a patient's jaw, filling a distal cavity with gold? Yet the nothing-but-gold-filling-men soon came to do that very thing. Rubber for plates was naughty, naughty,—why? Because dentists had to pay a license fee for using rubber. Today intelligent dentists often recommend rubber for a plate, overcoming the patient's preference for a gold base. Know a gold plate will not do. Recommend the gold inlay, yet if the patient can not afford it why—well, amalgam for this case will do very well, possibly. Tell me they're not a funny bunch, eh?

Yet to this day we find finicky patients, old bachelors often, imbued with the oldtime prejudice against rubber for plates. Must have a metal plate, whether or no. They guess they know a thing or two. No rubber plate for them, to salivate and ruin the health. Nor amalgam. The newer generation of dentists are altogether too smarty,—and so on.

But this isn't convention. Let's get aboard our train.

It may as well be understood that no reasonable dentist expects to come upon a lot of good, new things, at the convention, such as will revolutionize his practice. The three or four days' vacation counts for a whole lot, the meeting with old professional friends and acquaintances, the worthwhile hints he may gain in gossiping with them in the lobbies between sessions, at luncheon, while motoring, perhaps even at golf. These matters are uppermost in his thoughts weeks, months before he boards his train or speeds off with wife and sister in his car. He is going to have a change of scene, of association, is going to relax, forget office and patients alike. He is going to see worthwhile dental exhibits, other exhibits, new appliances ably demonstrated as to use, turn his attention to new ideas enunciated by leaders in the profession, and he has no objection whatever to listening to lectures and film talks on matters only remotely related to dentistry. Does n't the student in the university listen month after month to lectures on subjects having no practical relation whatsoever to the profession or the particular business he expects to devote his life to?

Don't be narrow-minded, fellows. The Greek and Latin the lawyer studied in college, only indirectly help him later on.

Some have regarded the film shows and lectures on subjects not directly associated with dentistry, as meretricious aids which might well be dispensed with. Not so. They give impetus to many a young dentist pondering the matter of adding a broader knowledge of general medicine to what the dental college has given him. It will not do to underrate their influence in creating among the dentist's patrons greater respect for his attainments. The general public comes to hear more of such matters than many suspect, and is impressed. A spice of humbuggery in it, you insinuate? Well, is n't there a spice of humbuggery in a lot of good things;—secret societies, women's clubs, health hints? As to this, however, we deny the imputation of humbuggery.

Come on, boys, old fellows. Save up for the next State Convention. Go without that fifteen dollar velour hat, that expensive radium-illuminated watch, that banquet lamp for the parlor. A small flashlight under your pillow beside your watch, answers every purpose of a radium-lighted watch. A fine felt hat is good enough for anybody. Save the money for that convention trip.

To begin with, you don't get a chance to sleep in a first-class hotel bed every week. It's a real pleasure to step out of bed into an inch deep of expensive rug, in the high-class hotel. You peer out of the windows upon sky-scrapers, upon everything wholly strange. You see similar surroundings in your own city, it may be, but not the same. The effect on your wearied nerves is like the music played in a hospital ward,—tonic, refreshing, rejuvenating.

Downstairs you go, find a lot of fellows you have not met for a year, going to breakfast. You join the procession, eat eggs, sweetbreads, hot cakes, drink a new brand of coffee. What a superb restaurant! Same kind of bacon, eggs, as you get at home, yet somehow better flavored, heaven-kissed, joy inspiring. Ain't you glad you came?

And the new faces of dentists you are introduced to! This is Dr. Orthodont, the world-renowned tooth-straightener from New York. Jolly fellow he is, too. You've been reading about him in dental journals for years and years. Had pictured him as rather squat, solemn visaged, silent, somewhat repelling. You find him tall, merry eyed, a good talker and an equally good listener. He sits beside you. You tell him, with some slight trepidation, answering his inquiry, that you are practicing in Blinkville, a village of fourteen hundred, and prepare to change your seat if he shows resentment. But he does not. Beams on you, says he'd like to trade expense accounts with you, as well as a bunch of his querulous patients who come in to an appointment after dancing half the night before. What joy it must be to have artesian well water, he half laments. And rustic patients without nerves. And auto drives with no worming through miles of congested streets, before you dare step on the gas for more than a two-miles-an-hour speed. Tells a funny story about a man disguising his "flivver" to look like a five thousand dollar car, entering it on a county fair race track, winning every race. It was such a rank counterfeit nobody could pass it. You spill coffee on your new silk tie, but what of it? You feel like kissing him. Such simplicity, genuineness. He is much your elder. After a few minutes while he is listening attentively to another prominent dentist talking, he reaches under the table and squeezes your hand softly. You feel a thrill of pleasure such as a girl seated in a grape arbor by moonlight feels, when her lover pats her hand.

As you sit smoking with the others, new friends and old, you begin to wonder if the convention itself has anything better in store for you than this. Begin to think of saving for the next National Convention, which you have never attended.

(What a delightful dream. Don't disturb me, boys.)

Ah, here we are in the convention hall. Convivial tremor in the air. Lot of good things smoking hot, soon to be served. "Who is that distinguished-looking man over there?" you ask, "That man in the gray suit." Somebody answers, "That's Dr. Upterdate, the celebrated bacteriologist, from Boston. Going to give us a screen lecture tonight. Live wire, you bet. Better come early, if you don't want to stand two hours."

Nobody seems to know this old man peering here and there among the already busy clinicians. Looks like an old sorehead. Wonder why he didn't stay home and play checkers with the druggist under his office. Now he is leaning over the heads of the three-deep circle of dentists in the clinic room adjoining, trying to get a glimpse of a new way of taking impressions. After five minutes of neck-craning he taps on the shoulder a young dentist trying to get a peep. Draws him aside, whispers audibly to him.

"Nothing in it, my young friend. Don't waste your time on that." Asks if he is blind, what's the matter with him that he does n't see the whiskers on the alleged new method. Shakes his head dismally. Hints darkly that the demonstrator is merely designing to win a reputation as a front-rank investigator and inventor. Wonders what dental supply house has hired him to help unload their shelves of a lot of junk.

Old man, you have a second think coming. Show us a dental supply man who is just so unscrupulous, and we'll show you one who has a child's judgment as to the intelligence of the average dentist. Where will you look for your man?

Old man slightly impressed by challenge. Winks knowingly, turns indifferently to find something else to criticize. Looks at his watch, guesses there's nothing new in anything forthcoming, for him. Pulls out a time table, want to know when the next train for Goosetown starts.

Hope he finds out. If he stays he is liable to get up during a session devoted to the latest phases of root-infection and rant about the upstart young graduate brimming with self-importance, who needs a sound spanking. And so on. Put him out, boys, some of you strong-armed fellows.

It is now two o'clock, and things are beginning to hum. Lecture on local anaesthesia by dentist of renown from Chicago. Don't frown disapprovingly; remember our own state celebrities are coming on, in due time. Mustn't be jealous. He tells us we have not yet found the quite satisfactory anaesthetic or method, but it is sure to come. Reviews the nine discarded or only half-approved methods. We have been reading our dental journals, but some things we must have missed, if we may believe what he tells us. This is a sort of free post-graduate course for heedless readers. Good enough.

The gentleman from Goosetown stands unobtrusively behind the last row of seats. He whispers that he thought he would take just a look in, his train not going for an hour yet. Seems in a querulous mood. Thinks the young graduate should be forbidden to bring his diploma with him to dental conventions, to flourish under his elders' noses, insulting them. Heard one, awhile ago saying to another, probably a classmate, that the old fellows who are always fault-finding, ought to comb the cobwebs out of their brains and try to learn some modern dentistry. If he had been twenty years younger, he would have landed a Dempsey sleep-producer on his jaw. Well, admitting that the youngster didn't mean him, exactly, yet it was an open insult to all the old timers like himself. No reverence for age and experience in this rapid age, on the part of the upstart young men. Would like to introduce a resolution covering the case, if opportunity offered. Doubts whether it would do any good. Youngsters in the majority here. Most of them beyond question, think dentists past fifty ought to be permanently shelved,—and so on.

We want to say, old man, that a few of the youngsters are eligible to that class too. Graduate in college, and are afraid to attempt a plaster impression! Believe all bicuspids have a single broad root, and spoil several double-rooted ones drilling between them for a pivot tooth pin. They seem not to have received any particular training as to that most important matter, the selection of the correct color for artificial dentures. Shirkers before and after graduation. Smoke up, you laggards.

Just a moment, Dr. Elderly from Goosetown, don't be in such a hurry about going. You may have a chance yet to get up when a discussion is on, and air your views. Stay over and see the end of this. If you don't care to make yourself so publicly conspicuous, some of us may arrange for a private convocation in some quiet corner of the hotel lobby. Get together an assortment of old dentists and young, not letting on what it is all for. There you may fire off your blast at your pleasure. Shouldn't so much wonder if some of the elders take sides with you. No hitting below the belt.

Yet it is the way of the world, today. Probably has so been during past ages. Sanguine youth takes little account of old age's experience. "All things have become new," youth interprets to mean that all past ideas and experiences count for naught. Whereas it may justly mean that the old has become incorporated with the latest-tested and approved, the result being a combination of the best in both old and new. Neither could possibly be spared.

Is not that about it? Both right, both wrong. If both of you old and young dentists would get together, smother all hostile impulses in a spirit of mutual desire to learn, it would be vastly to the advantage of both. What a profitable interchange of information would ensue.

This lecturer on anaesthetics has after all given us only a promise of something worthwhile coming before long. He has, however, given us something worthwhile in the way of hints what not to do, and that is something. Glad we listened. He has cleared away a lot of rubbish for us, at all events.

What is this coming on? Dr. Blank, another big University star, is to lecture on pyorrhea. Hold on, boys, don't run away. How do you know there's nothing in it? Hopeless case, you insist? Well, it does seem something like trying to cure a girl in love. The only way is to catch her before she falls in love. Treating an advanced case of pyorrhea is about as desperate an undertaking as fighting cockroaches in a wainscotted kitchen. In the end you have to tear out the wainscotting. Not always, but nearly.

Not many years ago we heard an eminent specialist whose name need not be mentioned, telling how to use his planers, what-nots. If treated teeth are unduly sensitive, after treatment, apply silver nitrate. If nothing helps, extract the teeth, he advised.

What the profession really needs is a description of pyorrhea specialist who will go to peoples' homes and scare them half to death as to their danger of contracting pyorrhea. Start treatment right there. Catch them at the breakfast table. Someone started a rumor not long ago, to the effect that cockroaches were suspected of causing cancer. (Propaganda by sellers of roach remedies, was it? Who knows.) Since the war we suspect propaganda in the apparently most innocent advertisement.

Hens have quit laying, short sugar crop. And strange to relate, a pyorrhea scare has for some time been in the air. Glad of it; keep it a-going. If the profession is sincere in making research along lines calculated to do away with itself, let us knock out pyorrhea first of all.

What the profession urgently needs today is some necromancer to merge dentistry and golf to some more practical purpose. Or tennis and dentistry. It is an age of wonders. Some think the telephone or the radio the most astounding of all. To the writer's conception neither approaches in the line of marvels the little needle following the almost indistinguishable tracings of the graphophone disc, evoking the music of band or orchestra, every characteristic tone of horn, clarinet, fife, drum. How can it be possible!

Oh for the glorious day when the dentist may invite his banker patient out to a full day of golf, summon little elves to be performing needed dental operations upon the unsuspecting patient swinging his clubs. Business and pleasure combined. Grateful banker, when he finds it out, sends check for sixty dollars. Same pleasing result of a forenoon of tennis, with a lady. Check next week—provided you have allowed her to win. Dental office dispensed with.

(Turn on the fan. Drive out these fumes.)

* * * Dinner at the hotel with numbers of representative men of our own and other states. Of course everybody talks shop. Curious devices withdrawn from vest pockets and handed round for inspection. Cunningly-devised screw matrix, which seems to meet all requirements. We must have one. No end of devices for matrix purposes in our drawers, in the office. Matrices are like other instruments dentists use. One is admirable in this respect but falls short in another equally important respect. The busy dentist is often impatient of delay caused by having to try this instrument, that one, a third, fourth, before finding what he instantly needs.

Give us a matrix that will fit anywhere, everywhere, screw itself on, stay where placed, reflect light. Somebody invent a little gnome to creep inside the matrix and see that it fits all around, to hold the first pellets of gold, ram them into retaining grooves, direct the plugger point, tell us where to shave the cavity to favor withdrawal of impression for inlay.

These trying deep cavities, where there's no time for making an inlay! Before adjusting matrix: smear cement, not too thick, over floor of cavity, filling the retaining grooves already cut. Ram three or four gold cylinders into grooves, forcing out most of the cement. When set condense gold, go ahead. No more tipping, loosening.

---

Thursday forenoon, nearing 12 o'clock. Dentists, some of them becoming restive, not quite so attentive to lecturer. Adjourn before long.

Reminds us of the last half of ninth inning at ball game. Two men out, two strikes on batter. Gang of fellows who think nothing of disturbing everybody, leaving their seats, making for the exits. You might think from the expression on their faces that they'd never had one particle of interest in baseball. Must get that car before the other fellow does.

Nothing so unmannerly as all that, in this instance, however. Want to visit with several old friends awhile, that is all. Afraid they'll be gone. Can not tell when they'll be going.

Now Dr. Stay-at-Home, we challenge you to deny that you have had a fine time, a profitable time. We ride a hundred miles with Chicago, Detroit, New York dentists, hear them review the lectures, tell what they approve, what disapprove. All this is often as profitable as the sessions in the convention hall. See how the fellows swarm around them, obstruct the aisle. Never mind; this is a specially chartered car. But do give the lady dentists at least a peep in.

Sing the old class songs, fellows, sing, sing. Yell if you must, that is, after a decorous manner. Admitting that we haven't learned enough new dentistry to revolutionize things in our practice, we have got two or three practical suggestions worth testing. We've met several dental celebrities, have made acquaintance of various others not so distinguished yet well worth knowing, have seen several new things in the dental exhibits we mean to investigate at our leisure, have gathered interest-reviving hints as to some old ones. Best of all; we have something new, refreshing, to think about for months to come.

Going again next year? You'd better believe we are.

